# Supplementary material for: Efficacy and Safety of 5-Aminolevulinic Acid Combined with Iron on Skeletal Muscle Mass Index and Physical Performance of Patients with Sarcopenia: A Multicenter, Double-Blinded, Randomized-Controlled Trial (ALADDIN Study)
Source: Nutrients. 2023 Jun 24;15(13):2866. doi: 10.3390/nu15132866 (PMC10343531; doi:10.3390/nu15132866)
Supplement: Supplementary file 1 [file nutrients-15-02866-s001.zip › nutrients-2442941-supplementary.pdf]

Supplementary Table S1. Participated medical institutions

Participated medical institution	Responsible investigator	Number of enrolled subjects
Yokufukai Hospital	Yasuko Abe	29
Juntendo Tokyo Koto Geriatric Medical Center	Hidenori Yoshii	21
Seino Internal Medical Clinic	Hiroaki Seino	14
Juntendo University Hospital	Yoshifumi Tamura	10
Japanese Red Cross Medical Center	Toru Hiyoshi	10
Misaki Naika Clinic	Nobuichi Kuribayashi	10
Saitama Medical University Hospital	Ikuo Inoue	6

Supplementary Table S2. Eligibility criteria

Inclusion criteria	Subjects who meet all of the following criteria are included in this study;<br>1. Male and female aged 65 years or older giving their consent<br>2. Male and female with a walking speed of less than 1 m/sec, men with a hand grip of less than 25 kg, or females with a hand grip of less than 20 kg<br>3. Subjects whose body mass index was less than 18.5 kg/m^2, males with lower leg circumference less than 34 cm, or females whose lower leg circumference was less than 33 cm<br>4. Males whose skeletal muscle mass index (SMI) measured by bioimpedance analysis (BIA) was less than 7.0 kg/m^2 or females whose SMI measured by BIA was less than 5.7 kg/m^2<br>5. Subjects who could provide written consent
Exclusion criteria	Subjects who fall into any of the following criteria are excluded from participating in the study;<br>1. Subjects with rheumatism<br>2. Subjects with dementia<br>3. Subjects with severe renal dysfunction (whose eGFRcreat was less than 30 mL/min/1.73 m^2) or subjects who were treated with dialysis<br>4. Subjects with severe hepatic dysfunction (whose AST or ALT was ≥3 times the upper limit of normal at the study execution medical institution)<br>5. Crippled subjects who needed legal representation to give consent<br>6. Subjects with a history of cerebral or myocardial infarction<br>7. Subjects with a pacemaker<br>8. Subjects allergic to 5-aminolevulinic acid<br>9. Subjects with porphyria

10. Subjects with drug hypersensitivity, such as allergy to iron

11. Subjects with other conditions that the responsible investigator or sub-investigators thought inappropriate for participation in the study

---

AST, aspartate aminotransferase; ALT, alanine aminotransferase; eGFRcreat, estimated glomerular filtration rate based on serum creatinine.

Supplementary Table S3. Observation schedule and items

	consenting	week 0 (baseline)*	week 4 ± 1 week	week 8 ± 2 weeks	week 12 ± 2 weeks
Eligibility information	○				
Background characteristics of subjects		○			
Blood pressure, calf circumference, and concomitant agents		○	○	△	○
Body composition		○	○	△	○
Physical activity (METs)		○			
6 minutes walking distance		△	△		△
Other physical function		○	○	△	○
Blood tests		○	○	△	○
QOL questionnaires		○			○
Adherence to study food intake		← ○ →			
Adverse event		← ○ →			

○: mandatory; △: optional

*Tests at week 0 (baseline) should be conducted before the initiation of the study food intake.

QOL: quality-of-life, METs: metabolic equivalents

Supplementary Table S4. Primary and secondary endpoints

Primary endpoint	Change in skeletal muscle mass index (SMI) measured by bioimpedance analysis (BIA) from baseline till the 12-week follow-up
Secondary endpoints	1. body weight
	2. BMI
	3. SMI measured by BIA
	4. basal metabolic rate
	5. hand grip
	6. lower leg circumference
	7. physical activity (METs)
	8. 6 minutes walking distance
	9. 5STS
	10. SPPB
	11. SF-36
	12. BDI
	13. EQ-5D-5L

BMI, body mass index; METs, metabolic equivalents; 5STS,5-repetition sit-to-stand test; SPPB, Short Physical Performance Battery; SF-36, MOS 36-Item Short-Form Health Survey; BDI, Beck Depression Inventory; EQ-5D-5L: EuroQol 5 Dimension-5 Level,
